# Subglottic spray via epidural catheter in awake tracheal intubation: A CARE-compliant case report

**DOI:** 10.1097/MD.0000000000041305

**Published:** 2025-02-14

**Authors:** Chao Chen, Junheng Chen, Weiqiang Chen, Chunming Guo, Yinzhou Zhan

**Affiliations:** a Department of Anesthesiology, Shantou Central Hospital, Shantou, Guangdong, China.

**Keywords:** anticipated difficult airway, awake tracheal intubation, epidural catheter spray, flexible bronchoscope, giant goiter

## Abstract

**Rationale:**

For anticipated difficult airways, awake tracheal intubation is strongly recommended by guidelines for its high success rate and safety. The key to successful awake tracheal intubation is effective airway topicalization. Currently available topicalization techniques have corresponding drawbacks and complications. Especially for subglottic topicalization, noninvasive techniques are not effective. Subglottic spray via epidural catheter with a flexible bronchoscope provided an effective and noninvasive technique.

**Patient concerns:**

A 73-year-old female patient with a history of giant goiter for 9 years suffered chest tightness and shortness of breath, and a 54-year-old male patient with a neck mass and hoarseness were scheduled for thyroidectomy.

**Diagnoses:**

Two cases of anticipated difficult airway due to compression by a giant goiter.

**Interventions:**

The subglottic area was topicalized via an epidural catheter with a flexible bronchoscope, and awake tracheal intubation was successfully performed.

**Outcomes:**

This technique was effective in eliminating airway reactions and hemodynamic fluctuations during awake intubation and improving the intubation success rate and patient tolerance.

**Lessons:**

This is the first report of subglottic topicalization via an epidural catheter with a flexible bronchoscope applied in cases of awake intubation in giant goiter. The epidural catheter spray is safe and effective in awake intubation of an anticipated difficult airway, and this noninvasive technique is an alternative option to transtracheal injection.

## 1. Introduction

Despite the low morbidity of substernal goiter, it is more likely to compress the airway associated with dyspnea.^[1]^ Patients often suffer from airway distortion and anticipated difficult airway due to the compression of goiter, which greatly increases the risk of the perioperative period. Inadequate management can lead to hypoxia, asphyxia, and even cardiac arrest.^[2]^ For difficult airway, awake tracheal intubation (ATI) is highly recommended by the American Society of Anesthesiologists (ASA) guidelines due to its 88% to 100% success rate.^[3]^

However, strong pharyngeal and airway reactions lead to fear and coughing as well as dramatic hemodynamic fluctuations in patients. The key to successful ATI is effective airway topicalization.^[4,5]^ Currently available topicalization techniques, such as mucosal atomization, ultrasonic nebulized inhalation, supraglottic nerve block, and transtracheal injection, are associated with drawbacks and complications.^[4,6–10]^ Moreover, invasive procedures are associated with risks of patient pain and discomfort, bleeding, and impalement, and usually need to be performed by experienced anesthesiologists.^[11]^ In addition, giant goiters covering the anterior neck make supraglottic nerve block and transtracheal injection impossible, while ultrasonic nebulization is unsatisfactory due to its uncertainty of inhalation concentration.^[11]^

Successful use of epidural catheter spray in morbidly obese patients or in those undergoing cervical spine surgery has been reported, but not in giant goiter.^[5,12,13]^ The difficulty in rapidly establishing an emergency invasive airway makes it more dependent on awake intubation. Herein, we reported 2 cases of anticipated difficult airway due to giant goiter, where subglottic topicalization was effectively administered via epidural catheter spray with flexible bronchoscope (FB), and awake intubation was successfully performed. Then we reviewed the related literature.

## 2. Case report

### 2.1. Case 1

A 73-year-old female patient (height 155 cm, weight 50 kg), with a history of giant goiter for 9 years, suffered chest tightness, shortness of breath, and sitting respiration suddenly in February 29, 2024. Because of coma, she was admitted to the emergency department 1 day later, with blood pressure 170/90 mm Hg, heart rate 125 bpm, and pulse oximetry 80%. Electrocardiogram (ECG) suggested frequent atrial premature and ST-segment marked depression; computed tomography (CT) showed the left side of a giant goiter, 80 × 75 mm, with the airway severely compressed, 3.11 mm at the narrowest point. The patient was diagnosed with hypoxemia and giant goiter, then transferred to the Intensive Care Unit. After noninvasive assisted ventilation, thyroid puncturing and drainage, and anti-infective treatment, the patient awoke with improved shortness of breath, and a repeat CT on day 6 showed that the narrowest of the airway was 7.96 mm, and thyroidectomy was performed on day 8.

Airway assessment was the most important part of preoperative evaluation. The patient revealed ASA III, mouth opening >3 cm, thyromental distance <6 cm, cervical lordosis <50°, Mallampati class III, and the cricothyroid membrane was not touched. Based on the evaluation, the patient was at risk for inability to ventilate after induction, difficult intubation, and difficult emergency invasive airway, which we assessed as a difficult airway and drew up a plan for awake intubation with a FB. The patient signed a consent form for epidural catheter spray as topicalization of awake intubation before the procedure. We recommended the extracorporeal membrane oxygenation (ECMO) backup plan to the patients during the preoperative conversation, that is, the ECMO tubes were pre-plumbed before the operation so that if endotracheal intubation fails, especially in anticipation of a difficult airway turning into an emergency airway, the ECMO could be activated quickly to avoid asphyxiation leading to sudden cardiac arrest. However, the patient declined this alternate option due to financial considerations. Thus, we invite the surgical team to stand by for an emergency airway. The subsequent step (plan B) is tracheotomy evaluation provided by the surgical team if 3 attempts at awake tracheal intubation fail. Awake tracheotomy will be performed by the surgical team after their professional assessment of the patient. If the surgical team’s assessment of the patient reveals that they cannot to do it, the surgery and anesthesia will be suspended, and the patient will be recommended to be transferred to a higher-level hospital.

**Figure 1.**
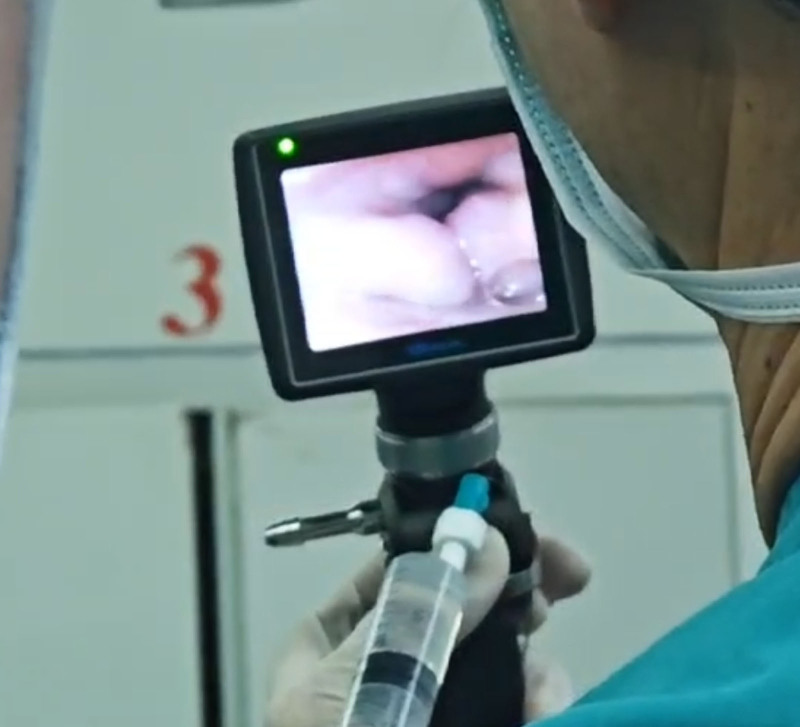
Epidural catheter with flexible bronchoscope topicalization.

**Figure 2.**
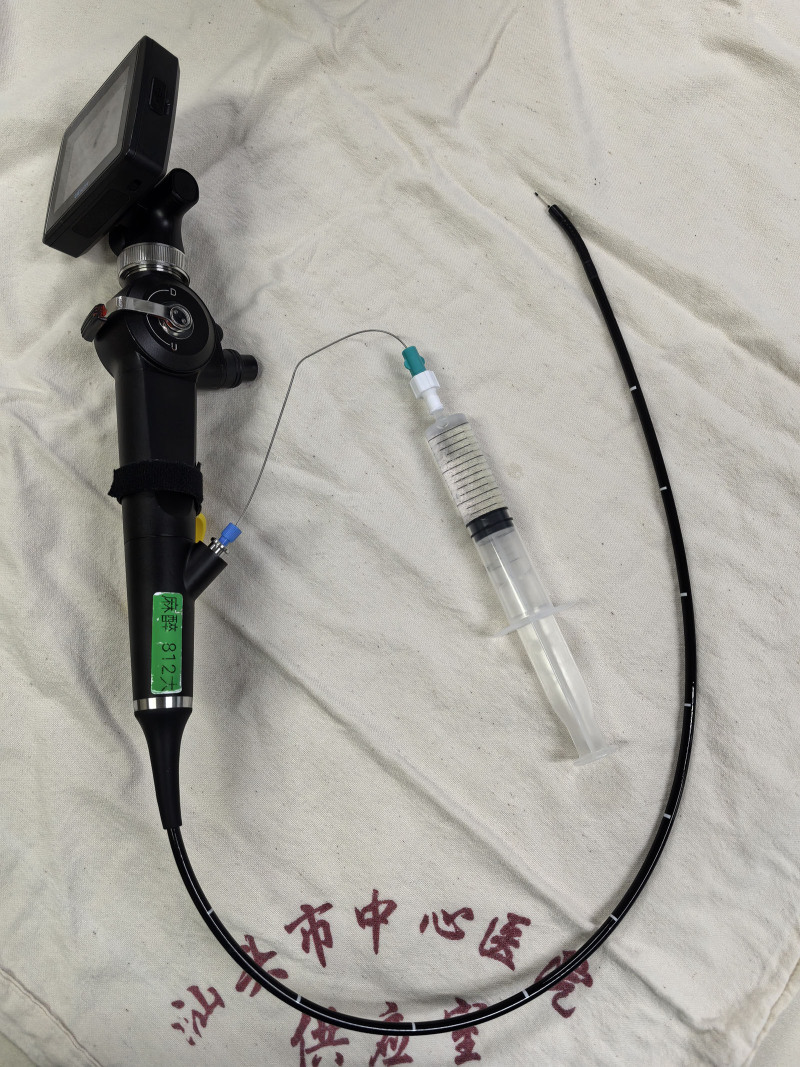
Epidural catheter get through the working channel of the flexible bronchoscope.

The patient was in the supine position and monitored by noninvasive blood pressure, electrocardiogram, and pulse oximetry after entering the operating theater. The oxygen was admitted by mask at a flow rate of 5 L/min, and ephedrine drops to constrict the nasal mucosal vasculature. After preoxygenation for 5 minutes, a spray bottle was used to spray 2% lidocaine, 5 to 10 sprays for each part of the patient’s oral cavity and tongue surface, tongue root, and pharynx sequentially, with an interval of 3 to 5 minutes. Then FB with the epidural catheter was used to topicalize the epiglottic, subglottic area, and trachea (Fig. 1). The epidural catheter was through the working channel of the FB, and the rear end of the epidural catheter was connected with a 2% lidocaine syringe to form a more fixed and adjustable direction of the nozzle (Fig. 2). The epidural catheter extended about 1 to 1.5 cm out of the working channel when topicalization, local anesthetic was sprayed out through lateral holes of the catheter, forming a large spray area and achieving effective topicalization. When topicalized the subglottic area, the bronchoscope was placed in the epiglottic area and the epidural catheter came out anteriorly, crossed over the vocal cords, and sprayed local anesthetic. This technique avoided touching the vocal cords and causing strong coughing, while providing effective topicalization of the proximal trachea. Considering that the patient was old and weak, and had low tolerance for the intubation, nasotracheal intubation was used to improve the patient’s comfort and tolerance, reduce the pain of the intubation process, increase the degree of cooperation of the patient, avoid the risk of biting of the flexible bronchoscope by the patient and increase the chances of success of the intubation. The tracheal tube (Shiley, ID 6.5, Covidien Ireland Limited) was intubated through the guidance of FB (Model PL-F420, diameter 4.2 mm, Zhejiang UE Medical Corp). After confirming the correct position of the tube, general anesthesia was induced and maintained, and respiration was maintained in volume-controlled ventilation mode. No adverse and unanticipated events happened during intubation. During the operation, the surgeon removed the goiter completely and explored the trachea without finding any tracheal tenderness or collapse. Then tracheal tube was removed after meeting the extubation criteria. The patient was transferred to the Intensive Care Unit after extubation, and to the general ward on the second postoperative day, and was discharged on the seventh postoperative day.

### 2.2. Case 2

A 54-year-old male patient was admitted to the emergency department in March 2021 with a neck mass and hoarseness. The patient had a history of a giant goiter for over a decade, with the sudden onset of hoarseness, dysphagia, and chest tightness half a month ago. There were no other accompanying symptoms, such as excessive sweating, irritability, lethargy, hand tremor, or numbness in the limbs. CT revealed a substantial enlargement of the thyroid gland on the left side and in the upper mediastinum, measuring 80 × 58 × 94 mm. This was accompanied by severe compression and narrowing of the airway, with a minimum diameter of 4.0 mm at the narrowest point, and paralysis of the vocal cords on the left side. ECG, blood tests, and chest X-rays did not reveal any notable abnormalities. The patient was referred to the Thoracic Surgery Department due to the extension of the mass to the retrosternum. Following confirmation of the diagnosis of a follicular adenoma of the thyroid gland, a combined thyroidectomy was performed.

The patient revealed body mass index of 21, ASA III, mouth opening >3 cm, Mallampati classification III, normal cervical lordosis, and the cricothyroid membrane and anterior cervical trachea were not palpable for cricothyroidotomy or tracheotomy. The patient was at risk of difficult intubation and potential difficulties with emergency invasive airway management. Therefore, we elected to utilize the FB awake intubation protocol and invited a thyroid surgeon to perform an emergency tracheotomy if necessary. The patient refused ECMO as an alternate option due to financial considerations, although we recommended it strongly. The patient signed a consent form for epidural catheter spray as topicalization of awake intubation before the procedure.

**Figure 3.**
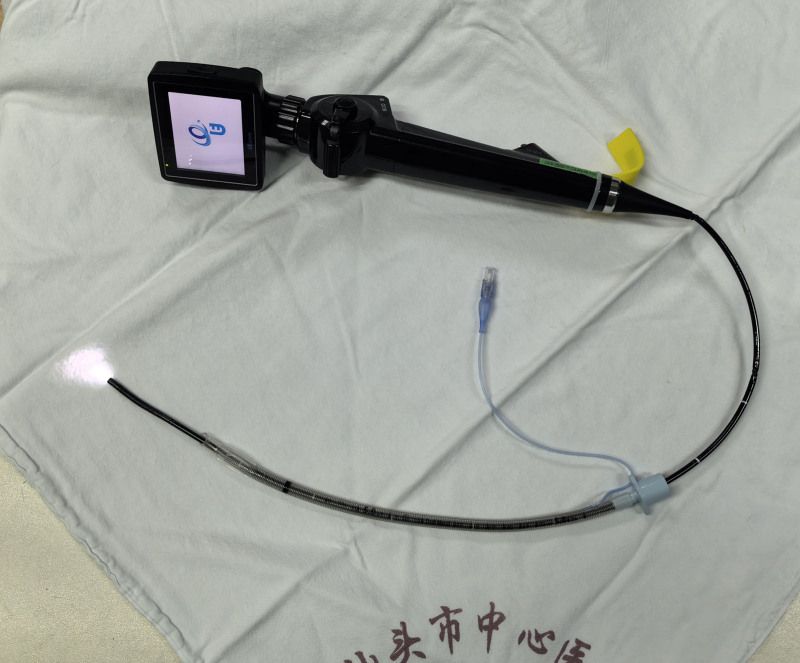
Flexible bronchoscope (PL-F420, Zhejiang UE Medical Corp, CHN).

**Figure 4.**
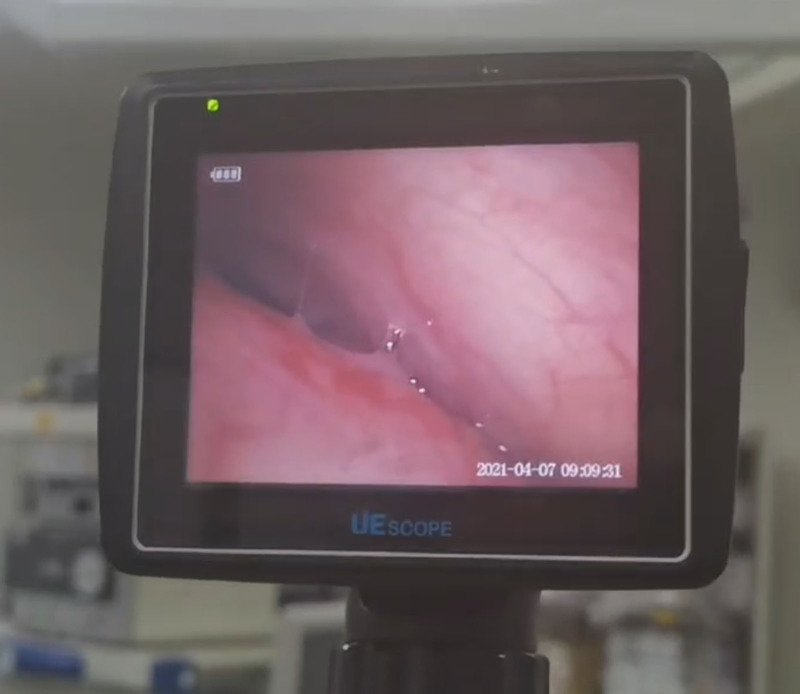
Compression of trachea under flexible bronchoscope.

**Figure 5.**
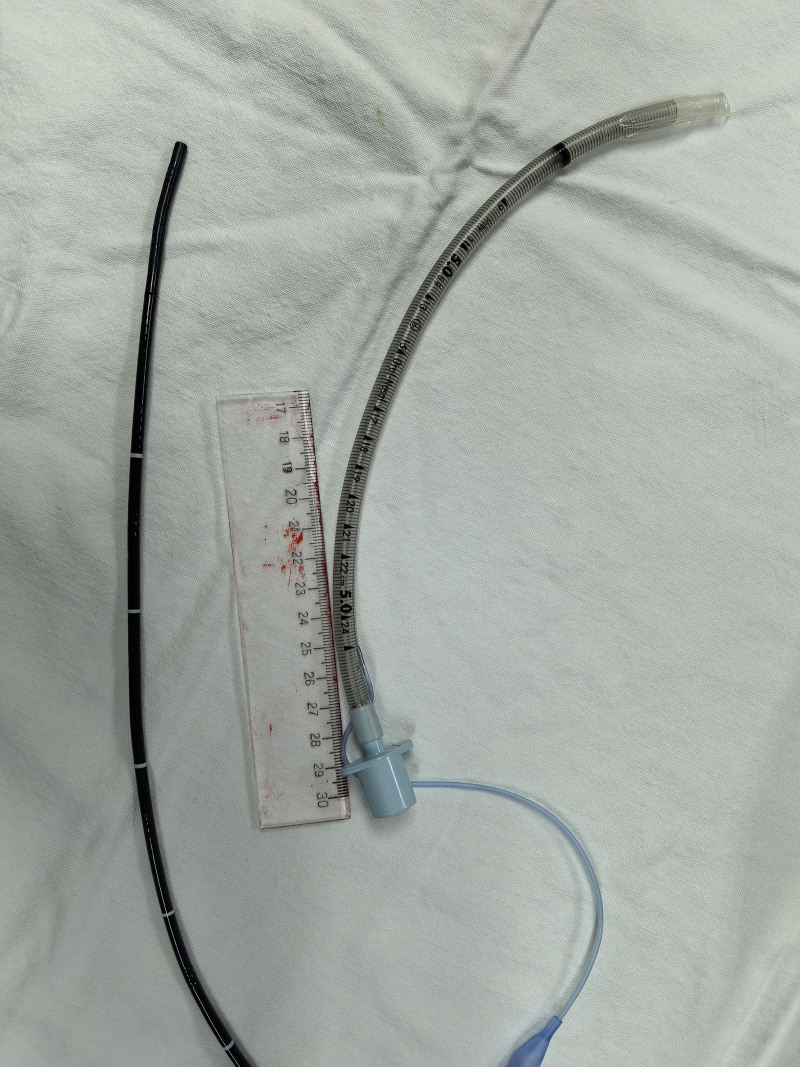
Reinforced tracheal intubation tube (Shiley, ID 5, Covidein, USA).

As previously outlined in case 1, the patient was admitted to the operative room with opening intravenous access, monitoring pulse oximetry, ECG, and blood pressure. In spite of the giant goiter, the patient did not have symptoms of airway obstruction and lay in the supine position. The ephedrine nasal dripping, sufentanil 5 µg infusion, and dexmedetomidine 0.5 µg/kg/h infusion for pumping were admitted. Following a 5-minute period of preoxygenation, the same topicalization procedure of case 1 was performed. In order to reduce the total time of the operation and the discomfort of the patients, for anesthesia in anticipation of a difficult airway, we usually do both nasal and oropharyngeal anesthesia at the same time, so that if one route fails a second one can be quickly attempted. With a high level of cooperation, the patient in case 2 was intubated by oral intubation after adequate oropharyngeal topical anesthesia. In the awake state, oral intubation allows the patient to be instructed to extend the tongue, open the mouth, and vocalize, which increases the operating space and increases the success rate of the first intubation. After taking into account the patient’s general status, level of patient’s cooperation, and the CT report, transoral intubation was used in case 2. During intubation guided by FB (Model PL-F420, Diameter 4.2mm, Zhejiang UE Medical Corp, Fig. 3), a dumbbell-shaped compression of the trachea at approximately 23 cm according to the incisors was observed (Fig. 4). After the FB had passed through the compressed segment, the inside diameter 5 reinforced tracheal tube (Shiley, ID5, Covidien Ireland Limited, Fig. 5), which had a scale of 25 cm and a total length of 27 cm, was rotated slowly to ensure that it had passed the segment and the tip did not inadvertently enter the bronchus. General anesthesia was then induced and maintained, with respiration maintained by volume-controlled ventilation mode. No adverse and unanticipated events happened during intubation. The surgeon’s intraoperative exploration of the trachea did not reveal any evidence of tracheal tenderness or collapse. Subsequently, following the resumption of spontaneous breathing by the patient in the postoperative period, the tracheal tube was removed and the patient was transferred back to the thoracic surgery ward, from which they were discharged on the fifth postoperative day.

## 3. Discussion

The 2 giant goiters were characterized by loss of airway and inability to establish an emergency invasive airway due to severe compression, which presented a significant challenge for the anesthesiologists in terms of airway management. Accordingly, in accordance with the recommendations of relevant guidelines, awake intubation was identified as the optimal approach for the anticipated difficult airway. Concurrently, the surgical team was instructed to stand by in preparation for the emergency invasive airway.

ATI allows for the preservation of spontaneous breathing, offers a safer alternative, and provides sufficient time to switch to other methods, including invasive emergency airway, ECMO, and so forth, in the event of failure. Furthermore, the success rate of intubation is high. Nevertheless, a strong airway response and fluctuations in hemodynamics can readily precipitate acute cardiovascular incidents in patients with hypertension or coronary artery disease. The patient must be conscious and able to collaborate with the operator, which is not feasible in patients with delirium, dementia, children, psychiatric disorders, or mental retardation. Intubation in an awake state intensifies patient distress and discomfort.

Notwithstanding the aforementioned disadvantages, awake intubation remains the most appropriate method of airway management in anticipation of a difficult airway when compared to patient safety. As topicalization techniques continue to evolve, these drawbacks are gradually becoming less of a limitation to awake intubation. It is noteworthy that the efficacy of awake intubation hinges on the administration of optimal subglottic topicalization. This not only mitigates airway responses and hemodynamic fluctuations but also alleviates patient anxiety and distress.^[14]^ The existing evidence indicates that ultrasonic nebulization, topical spray, and supraglottic nerve block can achieve satisfactory epiglottic anesthesia.^[11]^ Anesthesia under the vocal folds and proximal to the trachea poses difficulties for the anesthesiologist due to the obstruction of the vocal folds structure, and there is a large difference of the efficacy in anesthesia.

In the context of subglottic topicalization, techniques such as spray-as-you-go, ultrasonic nebulization, mucosal nebulization and transtracheal injection have become the predominant approaches. Empirical evidence has demonstrated that ultrasonic nebulization and mucosal nebulization are associated with greater patient comfort compared to spray-as-you-go. However, there is no discernible difference between nebulization and the s-technique with respect to hemodynamics, coughing and vomiting.^[15]^ Additionally, no differences in comfort scores, cough scores, total intubation time, or hemodynamics were observed between 2% lidocaine and 4% lidocaine in either spray or nebulization. However, the total amount of lidocaine administered and the blood concentration were higher with 4% lidocaine. No signs of toxicity or adverse effects were noted.^[16]^ In contrast, compared with 1% lidocaine, operators in the 2% lidocaine group achieved faster intubation times, higher operational satisfaction, and better patient tolerance. However, there was no difference in hemodynamic response between the 2 groups.^[17]^ The use of 2% lidocaine for subglottic topicalization in our case study yielded satisfactory anesthesia results, thereby providing further evidence in support of the efficacy of 2% lidocaine for this purpose. Conversely, the transtracheal injection is currently more commonly employed for subglottic topicalization, given the disadvantages of the nebulization technique, namely a prolonged anesthetic duration and an uncertain nebulized inhalation concentration. This results in a high incidence of coughing and vomiting during intubation, as well as an increased demand for lidocaine.^[10]^ Nevertheless, transtracheal injection represents an invasive anesthetic technique, which is associated with a number of potential complications, including tracheal mucosal bleeding and puncture of the esophagus. The ATI guidelines recommend that invasive techniques should be carried out by specialists. Consequently, this invasive technique is not widely available, which increases the barriers to anesthesiologists, and therefore requires training and experience. In the case of patients presenting with a difficult airway, the ASA guidelines recommend that tracheal management be performed by anesthesiologists with experience in this field. This results in a phenomenon known as “enrichment,” whereby trained and experienced doctors have greater opportunities to practice in the management of difficult airways, thereby becoming more skilled. This passive exclusion of younger doctors from this field deprives them of the chance to gain experience, which can have implications for their future practice. Furthermore, giant goiter may cover the trachea and larynx, and body markings may be difficult to palpate. While ultrasound can assist in identifying neck structures, the obstruction of the mass makes puncture impossible.

**Figure 6.**
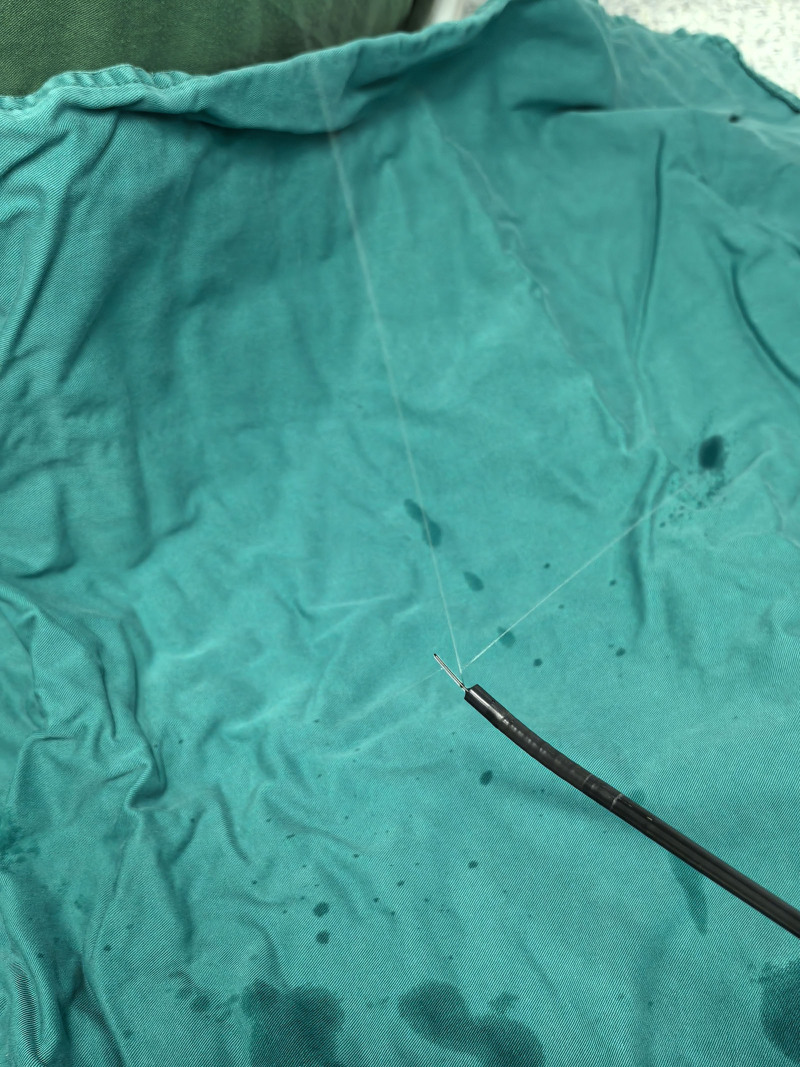
Multiple openings in the lateral wall of the epidural catheter makes spraying range wider.

Our case is the first application of FB combined with an epidural catheter for subglottic topicalization in giant goiter surgery. As mentioned previously, we obtained satisfactory results by spraying local anesthetics in the epiglottic, subglottic area and proximal airway through the epidural catheter under the guidance of FB. Compared with direct injection through the working channel of the bronchoscope, we believe that multiple openings in the lateral of the epidural catheter make the spraying range wider (Fig. 6). And under the guidance of the bronchoscope, local anesthetics can be a multidirectional adjustment of the spraying angle, and more accurately reach the target area. At the same time, the epidural catheter aperture is small, the drug spraying speed and dose are more controllable, and it is better tolerated by the patient because it is small to the water column and slight to the tracheal mucosa.^[13]^ Mccutchen et al compared the coughing response during ATI between epidural catheter technique and transtracheal injection, there was no significant difference between the 2 groups.^[5]^ Sethi et al demonstrated that the epidural catheter technique obtains better intubation conditions, less intubation time, and more accurate intubation results than nebulization and cricothyroid puncture methods, and less intubation time as well as less coughing.^[18]^ The British Difficult Airway Society guidelines for awake intubation recommend the epidural catheter technique as a remedy for inadequate local anesthesia, and our case also provides new evidence for the wider use of this technique.^[4]^ More importantly, we believe that the clinical value of this technique has been greatly underestimated and that this noninvasive technique avoids puncture-induced hemorrhage, mucosal injury, etc.^[19]^ Especially it is the presence of contraindications to transtracheal injection, such as anatomical abnormalities of the airway and infections of the cervical and mandibular spaces. Therefore, it is suggested that further study could focus on applying the epidural catheter technique as a first-line subglottic or epiglottic topicalization.

According to guideline recommendations, both emergency invasive airway and ECMO are alternate options for emergency airway. Previous studies have reported cases of loss of airway during difficult airway intubation and successful resuscitation with an emergency invasive airway. Xi and Rugnath reported successful experiences with ECMO for surgery of giant goiter, respectively.^[20,21]^ An emergency invasive airway was chose as a backup because of our thorough preoperative evaluation, the ability of the anterior end to cross the area of compression, and the use of a wire-type tracheal tube to provide effective support for the compressed airway area. We take a thorough preoperative assessment that the tip of the tracheal tube was able to cross the area of compression, and the use of a wire-type tracheal tube was able to provide effective support to the area of tracheal compression to ensure an open airway. We used a small dose of dexmedetomidine to relieve the patient’s nervousness without interfering with the patient’s respiratory function so that we were able to withdraw from anesthesia at any time and abort the procedure. Fortunately, both of our patients underwent ATI successfully and completed the surgery according to the plan, which is attributed to the robust effect of epineural topicalization, rich operating experience, and the cooperation of the patients. The success of ATI does not mean that the backup plan is ineffective; on the contrary, great importance was placed on a well-prepared backup plan and an emergency plan in difficult airway management. To illustrate, emergency plans such as emergency invasive airway as well as ECMO are also recommended even in patients with anatomical abnormalities such as giant goiter.

## 4. Conclusion

Subglottic spray via an epidural catheter with FB can achieve effective subglottic topicalization, enhance patient comfort and acceptance during ATI, and minimize airway responses and hemodynamic fluctuations during the procedure. This is the first report of epidural catheter technique for ATI in giant goiter patients, which confirms the safety and effectiveness for ATI procedures in anticipated difficult airways.

## Author contributions

**Conceptualization:** Chao Chen, Junheng Chen, Yinzhou Zhan.

**Supervision:** Chunming Guo, Yinzhou Zhan.

**Writing – original draft:** Chao Chen, Junheng Chen.

**Writing – review & editing:** Chao Chen, Junheng Chen, Weiqiang Chen, Yinzhou Zhan.
